# Neonatal outcomes according to different glucose threshold values in gestational diabetes: a register-based study

**DOI:** 10.1186/s12884-024-06473-4

**Published:** 2024-04-12

**Authors:** Kaisa Kariniemi, Marja Vääräsmäki, Tuija Männistö, Sanna Mustaniemi, Eero Kajantie, Sanna Eteläinen, Elina Keikkala, the Finnish Gestational Diabetes [FinnGeDi] study group, Anneli Pouta, Risto Kaaja, Johan G Eriksson, Hannele Laivuori, Mika Gissler

**Affiliations:** 1 Research Unit of Clinical Medicine, Medical Research Center Oulu, Oulu University Hospital and University of Oulu, Kajaanintie 50, 90220 Oulu, Finland; 2 Population Health Unit, Department of Public Health and Welfare, Finnish Institute for Health and Welfare, Helsinki, Finland; 3 NordLab, Oulu, Finland; 4 Faculty of Medicine, Research Unit of Translational Medicine, University of Oulu, Oulu, Finland; 5 Children’s Hospital, University of Helsinki and Helsinki University Hospital, Helsinki, Finland; 6 Department of Clinical and Molecular Medicine, Norwegian University of Science and Technology, Trondheim, Norway

**Keywords:** Gestational diabetes mellitus, Neonatal outcome, Neonatal hypoglycaemia, Oral glucose tolerance test, OGTT, IADPSG, NICE

## Abstract

**Background:**

Mild hyperglycaemia is associated with increased birth weight but association with other neonatal outcomes is controversial. We aimed to study neonatal outcomes in untreated mild hyperglycaemia using different oral glucose tolerance test (OGTT) thresholds.

**Methods:**

This register-based study included all (*n* = 4,939) singleton pregnant women participating a 75 g 2-h OGTT in six delivery hospitals in Finland in 2009. Finnish diagnostic cut-offs for GDM were fasting ≥ 5.3, 1 h ≥ 10.0 or 2-h glucose ≥ 8.6 mmol/L. Women who did not meet these criteria but met the International Association of the Diabetes and Pregnancy Study Groups (IADPSG) criteria (fasting 5.1–5.2 mmol/L and/or 2-h glucose 8.5 mmol/L, *n* = 509) or the National Institute for Health and Clinical Excellence (NICE) criteria (2-h glucose 7.8–8.5 mmol/L, *n* = 166) were considered as mild untreated hyperglycaemia. Women who met both the Finnish criteria and the IADPSG or the NICE criteria were considered as treated GDM groups (*n* = 1292 and *n* = 612, respectively). Controls were normoglycaemic according to all criteria (fasting glucose < 5.1 mmol/L, 1-h glucose < 10.0 mmol/L and 2-h glucose < 8.5 mmol/L, *n* = 3031). Untreated mild hyperglycemia groups were compared to controls and treated GDM groups. The primary outcome – a composite of adverse neonatal outcomes, including neonatal hypoglycaemia, hyperbilirubinaemia, birth trauma or perinatal mortality – was analysed using multivariate logistic regression.

**Results:**

The risk for the adverse neonatal outcome in untreated mild hyperglycemia was not increased compared to controls (adjusted odds ratio [aOR]: 1.01, 95% confidence interval [CI]: 0.71–1.44, using the IADPSG criteria; aOR: 1.05, 95% CI: 0.60–1.85, using the NICE criteria). The risk was lower compared to the treated IADPSG (aOR 0.38, 95% CI 0.27–0.53) or the treated NICE group (aOR 0.32, 95% CI 0.18–0.57).

**Discussion:**

The risk of adverse neonatal outcomes was not increased in mild untreated hyperglycaemia compared to normoglycaemic controls and was lower than in the treated GDM groups. The OGTT cut-offs of 5.3 mmol/L at fasting and 8.6 mmol/L at 2 h seem to sufficiently identify clinically relevant GDM, without excluding neonates with a risk of adverse outcomes.

**Supplementary Information:**

The online version contains supplementary material available at 10.1186/s12884-024-06473-4.

## Background

Gestational diabetes mellitus (GDM), defined as any degree of glucose intolerance with onset or first recognition during pregnancy [1], is associated with adverse neonatal outcomes, such as a higher risk of caesarean section [2–4], higher birth weight and risk of neonatal hypoglycaemia [4, 5] and hyperbilirubinemia [6–8].

Despite of these well-documented adverse outcomes, a consensus on the diagnostic criteria for GDM has yet to be reached. The International Association of Diabetes and Pregnancy Study Groups (IADPSG) recommended new diagnostic criteria for GDM in 2010 [9] after the Hyperglycemia and Adverse Pregnancy Outcome (HAPO) study [6] showed a linear relationship between maternal hyperglycaemia and unfavourable perinatal outcomes. The National Institute for Health and Clinical Excellence (NICE) did not adopt the guidelines introduced by the IADPSG because of insufficient evidence regarding the ability of the guidelines to improve pregnancy outcomes and yield cost savings [10]. The diagnostic cut-offs for GDM in Finland are based on the diagnostic criteria provided by the American Diabetes Association in 2003 [1], which differ slightly from both the IADPSG and NICE criteria (Table 1). Thus, it is possible to study the effects of different threshold glucose values on neonatal outcomes.

**Table 1:** Diagnostic cut-off values in the 75-g 2-h OGTT using different criteria

	IADPSG	NICE	Finnish current care guidelines
Fasting plasma glucose, mmol/L	5.1	5.6	5.3
1-h plasma glucose, mmol/L	10.0	-	10.0
2-h plasma glucose, mmol/L	8.5	7.8	8.6

*OGTT* Oral glucose tolerance test

*IADPSG* The International Association of Diabetes and Pregnancy Study Groups

*NICE* The National Institute for Health and Care Excellence

The aim of this study was to study neonatal outcomes in pregnancies that were not diagnosed as GDM by the criteria of the Finnish national guidelines and, thus, remained untreated but would have been diagnosed as GDM according to the criteria of the IADPSG or NICE. We hypothesised that for these women with mild untreated hyperglycaemia, as compared to normoglycemic controls or treated GDM, the risk of adverse neonatal outcomes would not be increased.

## Material and methods

### Study design

This register-based cohort study is part of the Finnish Gestational Diabetes study (FinnGeDi), which was initiated in 2009 after the former risk-based screening program for GDM was replaced with comprehensive screening [11, 12]. The registry data for this study were obtained from the Finnish Medical Birth Register (MBR), which contains information on all pregnancies resulting in a live birth or stillbirth at ≥ 22 gestational weeks or a birthweight ≥ 500 g. Data on pregnancy, delivery and neonatal outcomes were recorded, along with International Classification of Diseases (ICD) codes for diagnoses for the mother and child.

The registry data were combined with numerical oral glucose tolerance test (OGTT) data obtained from laboratory databases in two tertiary-level (North Ostrobothnia and Tampere) and four secondary-level (Satakunta, South Ostrobothnia, Kainuu and Southern Karelia) hospitals, each of which served a specific geographical area, in 2009. Deliveries in these hospitals accounted for 26.3% of all deliveries nationwide. These hospitals were selected for the study because they had numerical OGTT values available in their laboratory databases for the research. The selected population represents the Finnish pregnant population in 2009 well [11].

### Screening and diagnosis of GDM

All women, except those at very low risk of GDM (i.e. aged < 25 years, nulliparous, body mass index [BMI] of 18.5–24.9 kg/m^2^ and no family history of type 2 diabetes or aged < 40 years, multiparous without prior GDM or macrosomia and a BMI of < 25 kg/m^2^) were screened using 75-g 2-h OGTT after an overnight fast at 24–28 weeks of gestation [13]. For high-risk women, who were defined as having a history of GDM, a BMI ≥ 35.0 kg/m^2^, glucosuria during pregnancy, the use of glucocorticoids or polycystic ovary syndrome, an OGTT was performed at 12–16 weeks of gestation and repeated at 24–28 weeks of gestation if the test results were initially normal. If pre-pregnancy diabetes were suspected, an OGTT was performed immediately. If fasting glucose was ≥ 7.0 mmol/l or 2 h glucose was ≥ 11.1 mmol/l, pre-pregnancy diabetes was diagnosed. Venous samples were drawn into fluoride citrate tubes and analysed within 24 h in a local laboratory using commercial enzymatic assays. The involved laboratories were accredited under ISO15189:2012 standard and they had quality management systems. They performed regular internal quality control checks with controls of known concentrations and were also involved in external quality control schemes.

Gestational diabetes mellitus was diagnosed using national current care guidelines in Finland if any of the following cut-off values in the OGTT were met: ≥ 5.3 mmol/L, ≥ 10.0 mmol/L or ≥ 8.6 mmol/L for fasting, 1-h and 2-h plasma glucose values, respectively [13]. All women diagnosed with GDM were recommended to receive dietary and lifestyle counselling and instructions to self-monitor their glucose concentrations in public maternity clinics according to Finnish guidelines. Capillary glucose measurements were recommended to be taken after fasting in the morning and one hour after every main meal of the day (4 times per day). Insulin therapy was recommended if self-monitored capillary glucose concentrations exceeded 5.5 mmol/L after fasting or 7.8 mmol/L 1 h postprandial one to two times in a 1–2-week period, despite detailed diet and lifestyle counselling. The use of oral glucose-lowering agents was occasional, and they were not recommended by the current care guideline [13]. Women requiring medical treatment were recommended to be admitted to the outpatient maternity clinics of the hospitals serving their geographical areas.

### Study population and GDM groups

This study included singleton pregnant women who underwent an OGTT performed at 4–40 weeks of gestation during 2009. Women with pre-pregnancy type 1 or type 2 diabetes (ICD-10 codes E10–E11, E13, E14.9 or O24.0–O24.3) according to the MBR were excluded.

Women who fulfilled the diagnostic criteria of the IADPSG or NICE without meeting the Finnish criteria were untreated (Table 1). The treated groups met both the Finnish criteria for GDM and those of the IADPSG or NICE. Controls were normoglycemic according to all criteria. The untreated groups were compared to the normoglycaemic controls and to the treated GDM groups.

### Outcomes

The primary outcome was a composite of adverse neonatal outcomes comprising hypoglycaemia (ICD-10: P70.0–70.9), hyperbilirubinaemia (ICD-10: P59.0–59.9), birth trauma (Erb’s palsy [ICD-10: P14.0] or clavicle fracture [ICD-10: P13.4]) or perinatal mortality, which was defined as stillbirth or early neonatal death during the 7-day period after delivery. The secondary outcomes were the outcomes in the composite, which were analysed independently.

In all newborns exposed to GDM, glucose concentration was advised to be measured six times during the first 48 h [13]. Hypoglycaemia was treated with intravenous glucose if a single plasma glucose measurement was ≤ 1.4 mmol/L or when the initial glucose concentration was 1.5–2.5 mmol/L and remained ≤ 2.5 mmol/L after supplementary feeding. In addition to GDM, other potential indications of a need for neonatal glucose screening in asymptomatic newborns included preterm birth, a birth weight of < 2.5 kg or > 4.5 kg or maternal use of β-blockers. A diagnosis of hypoglycaemia was recorded in the MBR if a newborn required any interventions for the condition such as intravenous glucose, although there are no unified diagnostic criteria for neonatal hypoglycaemia in Finland.

### Covariates

Maternal age at delivery was determined. BMI (kg/m^2^) was calculated from self-reported pre-pregnancy height and weight and categorized into BMI groups according to World Health Organization criteria [14]. Parity was categorised according to the number of deliveries: primiparity, one (P1), two (P2), three (P3) or four or more previous deliveries (P4). Smoking status was categorized as smoking or not during pregnancy. Socioeconomic status was divided into upper white-collar, lower white-collar, blue-collar workers and others, including entrepreneurs, pensioners, stay-at-home-mothers and students. The use of insulin therapy during pregnancy was recorded. Maternal hypertension included chronic (ICD-10: O10.0-O12.1) and pregnancy-induced hypertension (ICD-10: O13) and pre-eclampsia (ICD-10: O14). Pregnancy-induced hypertension was defined as repeated blood pressure readings ≥ 140/90 mmHg after 20 weeks of gestation and pre-eclampsia was defined as repeated blood pressure readings ≥ 140/90 mmHg after 20 weeks of gestation and proteinuria (> 300 mg of protein in a 24-h urine collection or protein ≥ 2 + on a urine dipstick test). Data on the use of β-blockers were not available. Instead, maternal hypertension was used as a proxy variable. Labetalol, which is an unselective α- and β-blocker, is the preferred antihypertensive medication during pregnancy in Finland. The need for insulin therapy was based on the MBR (coded yes/no).

Birth weight (g), categorized as < 2.5 kg or > 4.5 kg; gestational age (weeks) at delivery; preterm birth, defined as delivery < 37 + 0 weeks of gestation; neonatal respiratory distress syndrome (ICD-10: P22.0); newborn discharge status (home or hospitalised) at the age of 7 days and duration of hospitalisation (days); the caesarean section rate and being large for gestational age (LGA), which was determined as a birth weight standard deviation (*SD*) score over the 90th percentile, were reported. The birth weight *SD* score was calculated from gestational age-adjusted birth weight according to standard growth curves in the Finnish population considering neonatal sex and maternal parity [15].

### Statistical analysis

Values are reported as mean (*SD*) for continuous variables and number and frequency (%) for categorical variables. To compare differences between the study groups, Pearson’s χ^2^ test was used for categorical variables, and the independent sample *t*-test was used for continuous variables. Logistic regression analyses were used to evaluate odds ratios (ORs), along with their 95% confidence intervals (CIs). To assess causality between GDM and adverse neonatal outcomes, a direct acyclic graph (DAG) was used (Supplementary figure). Based on the DAG, two models were formulated to adjust the results for potential bias. Model I included potential confounders (maternal age, pre-pregnancy BMI, parity, socioeconomic status and smoking status), and Model II included potential mediators (preterm birth, birth weight < 2.5 kg or > 4.5 kg and maternal hypertension), in addition to the confounders in Model I. The level of statistical significance was set at < 0.05 using a two-sided* p*-value. IBM SPSS Statistics version 27 (Armonk, NY, USA) was used for the analyses.

## Results

### Clinical characteristics

**Fig. 1:**
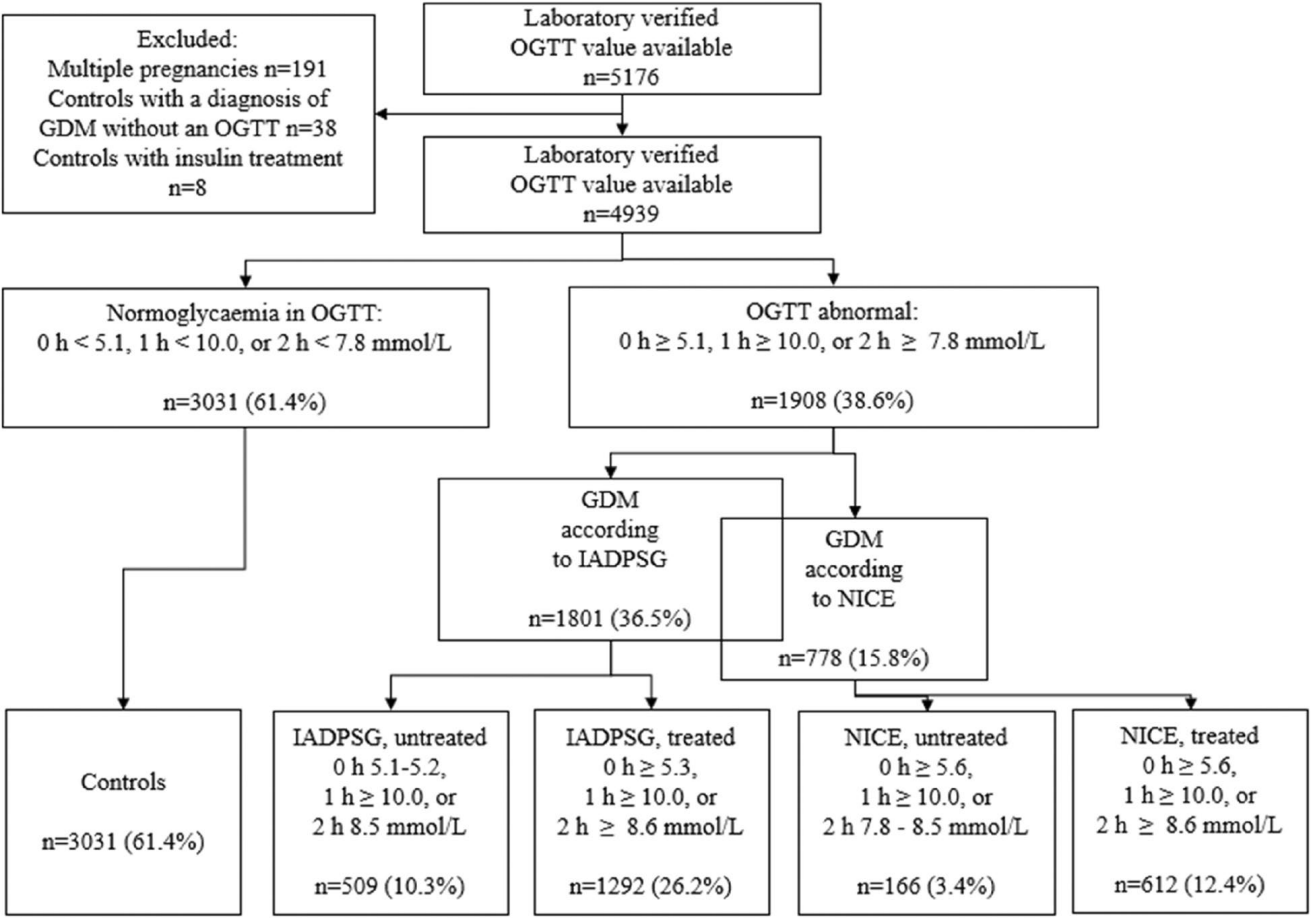
Flow chart of the study. GDM gestational diabetes mellitus, OGTT oral glucose tolerance test, IADPSG The International Association of Diabetes and Pregnancy Study Groups, NICE The National Institute for Health and Care Excellence.

After the exclusion of women with multiple pregnancies (*n* = 191), women in the control group treated with insulin (*n* = 8) and women with a diagnosis of GDM without an OGTT (*n* = 38), the total number of participants was 4,939 (Fig. 1). In the entire study population, 1,801 (36.5%) and 778 (15.8%) women had GDM according to the IADPSG and NICE criteria, respectively, and 1,292 (26.2%) had GDM according to the current care guidelines in Finland. In the IADPSG and NICE groups, 509 of 1,801 (28.3%) and 166 of 778 women (21.3%), respectively, were untreated because they did not meet the GDM criteria according to the current care guidelines in Finland. In the IADPSG group 1,292 (71.7%) women and 612 (78.7%) women in the NICE group received counselling and treatment for GDM. The control group included 3,031 (61.4%) women, who were normoglycemic according to all criteria. (Fig. 1, Table 1–2). Oral glucose tolerance tests were performed for 868 (17.6%) women before 24 weeks of gestation. 537 of the 1908 women (28.1%), who met any GDM criteria received diagnoses before 24 weeks of gestation. Insulin was started for 10.4% and 14.1% in the treated IADPSG and NICE groups, respectively. Detailed maternal characteristics are reported in Table 2, and neonatal characteristics are reported in Table 3.

**Table 2:** Maternal characteristics

	Controls	IADPSG				NICE			
		Untreated		Treated		Untreated		Treated	
			*pª*		*p*^b^		*pª*		*p*^b^
*N* (%)	3031 (61.4)	509 (10.3)		1292 (26.2)		166 (3.4)		612 (12.4)	
Maternal age, years, mean (*SD*)	29.4 (5.29)	30.1 (5.49)	0.009	30.5 (5.56)	0.157	30.0 (5.35)	0.127	30.8 (5.74)	0.110
Pre-pregnancy BMI, kg/m^2^, mean (*SD*)	25.6 (4.47)	27.3 (5.21)	< 0.001	28.4 (5.93)	< 0.001	25.9 (5.06)	0.456	28.8 (6.03)	< 0.001
BMI categorized, *n* (%)			< 0.001		< 0.001		0.600		< 0.001
< 18.5 kg/m^2^	61 (2.05)	5 (1.00)		16 (1.26)		5 (3.03)		6 (1.00)	
18.5–24.9 kg/m^2^	1380 (46.3)	180 (36.1)		376 (29.6)		72 (43.6)		171 (28.6)	
25.0–29.9 kg/m^2^	1092 (36.6)	190 (38.1)		425 (33.5)		58 (35.2)		185 (30.9)	
30.0–34.9 kg/m^2^	341 (11.4)	80 (16.0)		283 (22.3)		21 (12.7)		144 (24.1)	
≥ 35.0 kg/m^2^	107 (3.59)	44 (8.82)		170 (13.4)		9 (5.45)		92 (15.4)	
Parity, *n* (%)									
Primiparity	1461 (48.2)	213 (41.8)	0.008	483 (37.4)	0.086	83 (50.0)	0.690	242 (39.5)	0.017
P1	932 (30.7)	162 (31.8)	0.641	434 (33.6)	0.505	39 (23.5)	0.056	198 (32.4)	0.029
P2	382 (12.6)	74 (14.5)	0.252	205 (15.9)	0.515	28 (16.9)	0.120	88 (14.4)	0.461
P3	121 (4.0)	35 (6.9)	0.004	86 (6.7)	0.917	10 (6.0)	0.222	42 (6.9)	0.732
P4 or more	135 (4.5)	25 (4.9)	0.730	84 (6.5)	0.228	6 (3.6)	0.703	42 (6.9)	0.146
Smoking during pregnancy, *n* (%)	366 (12.1)	65 (12.8)	0.770	214 (16.6)	0.042	16 (9.6)	0.333	106 (17.3)	0.015
Socioeconomic status, *n* (%)			0.885		0.267		0.033		0.328
Upperwhite-collar worker	536 (17.7)	85 (16.7)	0.615	195 (15.1)	0.427	17 (10.2)	0.015	93 (15.2)	0.131
Lowerwhite-collar worker	1017 (33.6)	167 (32.8)	0.761	439 (34.0)	0.658	56 (33.7)	1.000	210 (34.3)	0.927
Blue-collar worker	399 (13.2)	68 (13.4)	0.944	211 (16.3)	0.129	31 (18.7)	0.047	99 (16.2)	0.482
Other	519 (17.1)	93 (18.3)	0.527	207 (16.0)	0.261	30 (18.1)	0.833	93 (15.2)	0.401
OGTT performed < 24 weeks	331 (10.9)	119 (23.3)	< 0.001	403 (31.2)	< 0.001	38 (22.9)	< 0.001	204 (33.3)	0.010
OGTT performed ^c^ / abnormal ^d^, weeks (SD)	26.7 (3.70) ^c^	25.8 (5.06) ^d^	< 0.001	24.7 (6.48) ^d^	< 0.001	26.7 (4.81) ^d^	0.999	24.9 (6.56) ^d^	< 0.001
Insulin treatment,* n* (%)	-	-	-	135 (10.4)	< 0.001	3 (1.8)	< 0.001	86 (14.1)	
Caesarean section, *n* (%)	453 (14.9)	98 (19.3)	0.015	271 (21.0)	0.437	32 (19.3)	0.148	136 (22.2)	0.457
Maternal hypertension, *n* (%)	225 (7.4)	49 (9.6)	0.089	151 (11.7)	0.213	16 (9.6)	0.364	82 (13.4)	

*IADPSG* The International Association of Diabetes and Pregnancy Study Groups

*NICE* The National Institute for Health and Care Excellence

*SD* Standard deviation

*BMI* Body mass index, *OGTT* Oral glucose tolerance test

*ªp*-value between the GDM group and controls

^b^*p*-value between the treated and non-treated GDM groups

^c^Weeks of gestation for the last OGTT performed for controls

^d^Weeks of gestation for abnormal OGTT according to defined criteria

Parity was categorised according to the number of deliveries: primiparity, one (P1), two (P2), three (P3) and four or more previous deliveries (P4)

Upperwhite-collar workers included administrative, managerial, professional and related occupations; lowerwhite-collar workers included administrative and clerical occupations; blue-collar workers included manual labourers; and other included individuals who did not fit any of the above categories, including students, pensioners and self-employed

A diagnosis of maternal hypertension included ICD-10 codes O10-O12.1 and O13-O14

**Table 3:** Neonatal outcomes in the untreated and treated GDM groups using the IADPSG and NICE criteria

	Controls	IADPSG				NICE			
		Untreated		Treated		Untreated		Treated	
			*pª*		*p*^b^		*pª*		*p*^b^
*N* (%)	3031 (61.4)	509 (10.3)		1292 (26.2)		166 (3.4)		612 (12.4)	
Composite adverse neonatal outcome, *n* (%)	279 (9.2)	51 (10.0)	0.564	287 (22.2)	< 0.001	20 (12.0)	0.272	164 (26.8)	< 0.001
Hypoglycaemia, *n* (%)	74 (2.4)	17 (3.3)	0.288	187 (14.5)	< 0.001	6 (3.6)	0.439	106 (17.3)	< 0.001
Hyperbilirubinaemia, *n* (%)	172 (5.7)	32 (6.3)	0.607	108 (8.4)	0.144	9 (5.4)	1.000	62 (10.1)	0.068
Birth trauma, *n* (%)	39 (1.3)	4 (0.8)	0.393	21 (1.6)	0.189	3 (1.8)	0.723	13 (2.1)	1.000
Perinatal mortality, *n* (%)	6 (0.2)	1 (0.2)	1.000	5 (0.4)	0.682	2 (1.2)	0.061	4 (0.7)	0.613
Birth weight, g, mean (*SD*)	3572.7 (525.7)	3632.9 (543.5)	0.017	3561.5 (562.3)	0.014	3499.9 (603.2)	0.130	3539.9 (623.5)	0.461
Birth weight < 2.5 kg, *n* (%)	89 (2.9)	18 (3.5)	0.483	49 (3.8)	0.890	9 (5.4)	0.098	34 (5.6)	1.000
Birth weight > 4.5 kg, *n* (%)	94 (3.1)	21 (4.1)	0.279	36 (2.8)	0.177	6 (3.6)	0.818	21 (3.4)	1.000
Birth weight *SD* score, mean (*SD*)	-0.047 (1.01)	0.12 (1.05)	< 0.001	0.072 (1.08)	0.434	0.011 (1.05)	0.476	0.12 (1.19)	0.293
LGA, *n* (%)	267 (8.8)	65 (12.8)	0.005	147 (11.4)	0.417	22 (13.3)	0.069	82 (13.4)	1.000
Gestational age at delivery in weeks, mean (*SD*)	39.9 (1.57)	39.8 (1.60)	0.154	39.5 (1.81)	< 0.001	39.5 (1.93)	0.002	39.2 (2.01)	0.185
Preterm birth < 37 weeks, *n* (%)	114 (3.8)	21 (4.1)	0.707	78 (6.0)	0.135	12 (7.2)	0.037	49 (8.0)	0.751
Neonatal respiratory distress syndrome, *n* (%)	15 (0.5)	3 (0.6)	1.000	9 (0.7)	1.000	1 (0.6)	1.000	5 (0.8)	0.293
Newborn discharge status aged 7 days, *n* (%)									
Home	2895 (95.5)	482 (94.7)	0.423	1195 (92.5)	0.099	152 (91.6)	0.025	547 (89.4)	0.470
Hospitalised	130 (4.3)	25 (4.9)	0.558	91 (7.0)	0.110	11 (6.6)	0.170	61 (10.0)	0.227
Hospitalisation in days, mean (*SD*)	3.1 (2.61)	3.2 (1.37)	0.514	3.3 (2.09)	0.157	3.3 (1.45)	0.425	3.5 (2.71)	0.315

*GDM* Gestational diabetes mellitus

*IADPSG* The International Association of Diabetes and Pregnancy Study Groups

*NICE* The National Institute for Health and Care Excellence

*ªp*-value between the GDM group and controls

^b^*p*-value between the treated and untreated GDM groups

*SD* Standard deviation

The composite adverse neonatal outcome included hypoglycaemia, hyperbilirubinaemia, birth trauma or perinatal mortality

Birth trauma included Erb’s palsy or clavicle fracture

*LGA* Large for gestational age, defined as having a birth weight *SD* score > 90th percentile

### Outcomes

**Fig. 2:**
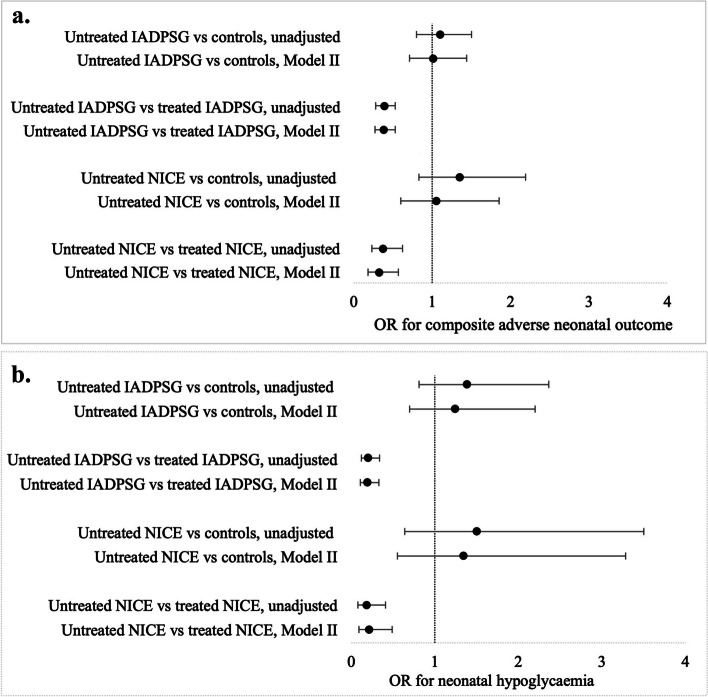
**a** Odds ratios (ORs) and 95% confidence intervals (CIs) for the composite adverse neonatal outcome in the treated and untreated IADPSG and NICE groups versus the control group. **b** ORs and 95% CIs for hypoglycaemia in the treated and untreated IADPSG and NICE groups versus the control group. ORs odds ratios, CIs confidence intervals, GDM gestational diabetes mellitus, IADPSG The International Association of Diabetes and Pregnancy Study Groups, NICE The National Institute for Health and Care Excellence. The composite adverse neonatal outcome included neonatal hypoglycaemia, hyperbilirubinaemia, birth trauma or perinatal mortality. Birth trauma included clavicle fracture or Erb’s palsy. Model II: adjusted for maternal age, pre-pregnancy body mass index (BMI), parity, smoking status, socioeconomic status, preterm birth < 37 weeks of gestation, birth weight > 4. 5 kg or < 2.5 kg and maternal hypertension (pre-pregnancy or pregnancy-induced)

The rate of the composite adverse neonatal outcomes did not differ between normoglycaemic controls (9.2%) and the untreated IADPSG group (10.5%, adjusted odds ratio [aOR] 1.01, 95% confidence interval [CI] 0.71–1.44) or the untreated NICE group (12.0%, aOR 1.05, 95% CI 0.60–1.85). In the untreated hyperglycaemia groups the rates of the composite adverse neonatal outcomes were lower than in the respective treated groups: 10.5% vs 22.2% (aOR 0.38, 95% CI: 0.27–0.53) in the IADPSG group and 12.0% vs 26.8% (aOR 0.32, 95% CI 0.18–0.57) in the treated NICE group (Tables 3–4, Fig. 2a). The rate of neonatal hypoglycaemia did not differ between the untreated groups and normoglyceamic women (untreated IADPSG: 3.3% vs 2.4% in the control group, aOR 1.24, 95% CI 0.70–2.20; untreated NICE: 3.6% vs 2.4%, aOR 1.34, 95% CI 0.55–3.28). Rates of neonatal hypoglycaemia were lower in the untreated hyperglycaemia groups as compared to the treated groups: 3.3% vs. 14.5% (aOR 0.19, 95% CI 0.11–0.33) in the IADPSG group and 3.6% vs. 17.3% (aOR 0.21, 95% CI 0.09–0.49) in the NICE group (Tables 3–4, Fig. 2b). There were no differences in the frequency of hyperbilirubinaemia between the mild untreated hyperglyceamia and controls, and slightly lower prevalence of hyperbilirubinaemia in mild hyperglyceamia compared to treated NICE group after adjusting for confounders. No between-group differences were observed in the rate of perinatal mortality or birth trauma.

**Table 4:** Regression analyses of neonatal outcomes in untreated groups compared to controls or the treated groups

		Untreated IADPSG		Untreated NICE	
Comparison		Controls	Treated IADPSG	Controls	Treated NICE
Composite adverse neonatal outcome	Unadjusted OR	1.10 (0.80–1.50)	0.39 (0.28–0.53)	1.35 (0.83–2.19)	0.37 (0.23–0.62)
	Model I	1.07 (0.77–1.48)	0.39 (0.28–0.54)	1.26 (0.75–2.09)	0.35 (0.20–0.60)
	Model II	1.01 (0.71–1.44)	0.38 (0.27–0.53)	1.05 (0.60–1.85)	0.32 (0.18–0.57)
Hypoglycaemia	Unadjusted OR	1.38 (0.81–2.36)	0.20 (0.12–0.34)	1.50 (0.64–3.50)	0.18 (0.08–0.41)
	Model I	1.32 (0.76–2.31)	0.20 (0.12–0.33)	1.59 (0.68–3.76)	0.21 (0.09–0.49)
	Model II	1.24 (0.70–2.20)	0.19 (0.11–0.33)	1.34 (0.55–3.28)	0.21 (0.09–0.49)
Hyperbilirubinaemia	Unadjusted OR	1.12 (0.76–1.65)	0.74 (0.49–1.11)	0.95 (0.48–1.90)	0.51 (0.25–1.03)
	Model I	1.11 (0.75–1.67)	0.75 (0.49–1.14)	0.87 (0.42–1.81)	0.44 (0.20–0.95)
	Model II	1.04 (0.67–1.63)	0.79 (0.51–1.23)	0.61 (0.26–1.40)	0.41 (0.17–0.98)
Perinatal mortality	Unadjusted OR	1.0 (0.12–8.26)	0.51 (0.06–4.35)	6.15 (1.23–30.7)	1.85 (0.34–10.00)
	Model I	-		4.02 (0.44–36.4)	1.05 (0.10–11.11)
	Model II	-		3.69 (0.24–57.9)	-
Birth trauma	Unadjusted OR	0.61 (0.22–1.71)	0.41 (0.16–1.41)	1.41 (0.43–4.62)	0.85 (0.24–3.03)
	Model I	0.58 (0.20–1.65)	0.46 (0.16–1.37)	1.46 (0.44–4.83)	0.86 (0.17–2.27)
	Model II	0.57 (0.20–1.63)	0.45 (0.15–1.37)	1.51 (0.46–5.02)	0.65 (0.17–2.44)

*IADPSG* The International Association of Diabetes and Pregnancy Study Groups

*NICE* The National Institute for Health and Care Excellence

*OR* Odds ratio

The composite adverse neonatal outcome included neonatal hypoglycaemia, hyperbilirubinaemia, birth trauma or perinatal mortality

Birth trauma included clavicle fracture or Erb’s palsy

Model I: adjusted for maternal age, pre-pregnancy BMI, parity, smoking and socioeconomic status; Model II: adjusted for the variables in Model I, preterm birth: < 37 weeks of gestation, very high birth weight: > 4.5 kg, very low birth weight: < 2.5 kg, and maternal hypertension (pre-pregnancy or pregnancy-induced)

## Discussion

### Main findings

As hypothesised, the risk of adverse neonatal outcomes in the untreated groups representing untreated mild maternal hyperglycaemia was similar to that in normoglycemic controls. The risk of neonatal morbidity in the untreated GDM groups was also lower as compared to the treated groups. These novel findings suggest that mild hyperglycemia, which is defined as GDM according to the IADPSG or NICE definitions, does not have a negative impact on neonatal outcomes in clinical practice.

Several previous studies show that higher maternal blood glucose levels are associated with adverse neonatal outcomes, which was demonstrated first in the HAPO study [6] and later corroborated in other studies [8, 16–20]. However, the question of adequate cut-offs remains widely debated. The potential of diagnosing and treating even mild hyperglycaemia, as applied in the IADPSG criteria to prevent adverse neonatal outcomes has been discussed in the literature [21–26]. Because of the variable diagnostic criteria used for GDM and thresholds for treating GDM, studies have produced inconsistent results. Most have found that the treatment of mild hyperglycaemia reduced adverse neonatal outcomes [23–27], while others have not [21, 22]. In studies in which a favourable treatment effect was found, the participants would have met the diagnostic criteria for GDM used in Finland and, therefore, received treatment. In only one study [22] did the OGTT criteria for GDM match those used in the untreated IADPSG group of our study, and they found no differences in neonatal outcomes when comparing the untreated and their treated counterparts. Our group and others have found that mild hyperglycaemia is associated with slightly higher birth weights and caesarean section rates [4, 6, 16, 26, 28]. The treatment of GDM could potentially reduce the risk of these outcomes [25–27], but based on our findings, adopting the IADPSG criteria in Finland would not result in clinically significant improvements in adverse neonatal outcomes such as hypoglycaemia. Simultaneously, there would be 39% more GDM diagnoses, as the incidence of GDM in the screened population was 26.2% when using the Finnish cut-offs and 36.5% when using the IAPDSG cut-offs. Therefore, according to our findings, the current diagnostic criteria for GDM in Finland with slightly higher fasting (5.3 vs 5.1 mmol/L) and 2-h (8.6 vs 8.5 mmol/L) OGTT cut-offs than the IADPSG criteria are sufficient to identify women at risk of adverse neonatal outcomes.

We found that untreated mild hyperglycaemia is associated with a 2–threefold lower risk of the adverse neonatal outcomes as compared to the treated groups. This finding suggests that those with mild hyperglycemia, even if it is untreated, have a much lower risk of neonatal adverse outcomes, especially hypoglycemia, than those with treated GDM. In contrast, we conclude that those with treated GDM still have increased risk of adverse neonatal outcomes, which is in line with several previous studies [16–20]. The risk of adverse outcomes, such as macrosomia and birth traumas, can be decreased with GDM treatment, but not all of them can be prevented [26, 27]. Moreover, according to randomized controlled trials, the treatment of mild GDM does not seem to prevent neonatal hypoglycemia [25, 27].

The relatively high rate of adverse neonatal outcomes in the treated GDM groups of our study raises the question of whether the management of GDM was successful during the study period. Potential explanations include the relatively high target fasting value for the self-monitoring of blood glucose, which is < 5.5 mmol/L in the Finnish guidelines, whereas in some international guidelines the target concentration is < 5.3 mmol/L [10, 29]. In our study the rate of insulin use was low in the treated groups (10.4% and 14.1% in the treated IADPSG and treated NICE groups, respectively). Worse glycemic control is associated with more adverse neonatal outcomes and both the recommended target values for the self-monitoring of blood glucose and the rate at which the targets are met influence outcomes [30–32]. However, in the TARGET trial, the stricter threshold for fasting plasma glucose concentration (< 5.0 mmol/L), as compared to less strict threshold (< 5.5 mmol/L) for glycaemic control in women with GDM, resulted in a similar incidence of LGA infants, neonatal hypoglycaemia, hyperbilirubinaemia, the need for respiratory support and maternal outcomes such as preeclampsia, the need for the induction of labour and caesarean births. The reduced incidence of neonatal birth traumas and mortality and increased serious maternal obstetric complications such as haemorrhage and embolism were observed in the group with a stricter threshold [30]. In another study, shorter time to achieve glycaemic controls and a longer duration in an optimal glycaemic control resulted in fewer perinatal complications [31]. In our study, as it is register-based, we were not able to record glyceamic control during the pregnancies.

In Finland OGTTs are recommended at 12–16 weeks of gestation for high-risk women [13]. This differs from the other recommendations. However, the IADPSG recommends a fasting glucose screening for high-risk women prior 24 weeks. If fasting glucose is 5.1–6.9 mmol/L, a diagnosis of GDM is made, and if fasting glucose exceeds 6.9 mmol/L, a diagnosis of overt diabetes is made [9]. Therefore, to our understanding, a diagnosis of GDM can be made before 24 weeks. Validated cut-offs for early-pregnancy OGTTs are not yet accepted. However, there is an intention to engage in earlier screening for GDM and evidence, which favours slightly higher cut-offs than in the IADPSG recommendation [33]. We decided not to exclude women who were diagnosed with GDM before 24 gestational weeks, because this would have distorted our study population, as we would have excluded one-fourth of all GDM women and women who have risk factors for adverse outcomes [11].

Gestational diabetes mellitus leads to a hyperglycaemic intrauterine environment, which results in a transient amplified insulin response in the newborn, causing hypoglycaemia. This, combined with the neonatal glucose screening used for GDM-exposed newborns explains the high frequency of neonatal hypoglycaemia among the treated GDM groups in our study. We adjusted for other potential risk factors for neonatal hypoglycaemia, which did not significantly attenuate the risk associated with GDM and, therefore, are unlikely to mediate the association between GDM and neonatal hypoglycaemia in this study. The risk of neonatal hypoglycaemia seems to be greater at higher concentrations of maternal blood glucose [16, 25–27], and this explains why the rate of neonatal hypoglycaemia was similar in the untreated GDM groups and controls in our study. However, newborns in these groups did not undergo routine postnatal glucose testing which could have led to undiagnosed cases of neonatal hypoglycaemia. On the other hand, all newborns presenting with symptoms were tested for potential hypoglycaemia and therefore all cases of symptomatic neonatal hypoglycaemia were most likely identified.

### Strengths and limitations

The strengths of this study include the use of well-defined and comprehensive population-based register data, as the MBR includes information on all births in Finland. The validity of the data in the MBR has been demonstrated to be high [34]. The public maternity clinic system in Finland is free of cost, statutorily organised and comprehensive because 99.7% of women attend these clinics during pregnancy [35]. By collecting numerical data from the OGTTs we were able to divide the participants into GDM groups according to the IADPSG and NICE criteria and evaluate neonatal outcomes in the untreated groups. We were able to adjust for important confounding factors and potential mediators.

As Finnish national guidelines do not suggest screening very low-risk women for GDM with OGTTs, these women were excluded from our study. Oral glucose tolerance tests were performed for 42.6% and 71.0% of pregnant women in Finland in 2009 and 2021, respectively. Since the study period, the key risk factors for GDM, advanced age and higher pre-pregnancy BMI have become more prevalent in terms of explaining the increase in the OGTT screening rate [36]. Also, the use of metformin has been accepted in updated national guidelines. These changes may have some effects on the interpretation of the results. Since this is a register-based study, we were unable to verify whether GDM was managed according to the recommended guidelines. The untreated NICE group was small (*n* = 166) and thus, the power to detect small differences in the frequencies of adverse neonatal outcomes in the NICE group versus the control group was limited. There may be some uncertainty involved in the detection of neonatal hypoglycemia due to a lack of nationally unified diagnostical criteria. In addition, misclassification bias in the treated GDM groups could have potentially increased the prevalence of specific outcomes in these groups, as those treated for GDM are screened for neonatal hypoglycemia more likely than others. Finally, this study lacks data on the glycaemic control of the study participants, which could offer an explanation as to why the treated GDM groups had higher rates of unfavourable neonatal outcomes as compared to those with mild untreated hyperglycaemia.

### Conclusions

Untreated mild hyperglycaemia was not associated with poor neonatal outcomes. The diagnostic criteria used in Finland seem to sufficiently identify clinically relevant GDM, without excluding neonates with a risk of adverse outcomes.

### Supplementary Information

**Supplementary Material 1. **

## Data Availability

The data are not open access according to the Finnish legislation. The use of registry data requires permission from national registry authorities. Metadata of the current study are available from the corresponding author on reasonable request.
