# Supplementary material for: Neonatal outcomes according to different glucose threshold values in gestational diabetes: a register-based study
Source: BMC Pregnancy Childbirth. 2024 Apr 12;24:271. doi: 10.1186/s12884-024-06473-4 (PMC11010296; doi:10.1186/s12884-024-06473-4)
Supplement: Supplementary file 1 — Supplementary Material 1. [file 12884_2024_6473_MOESM1_ESM.docx]

**Figure legend**
**Supplementary figure.** DAG to assess causality between GDM and adverse neonatal outcomes.


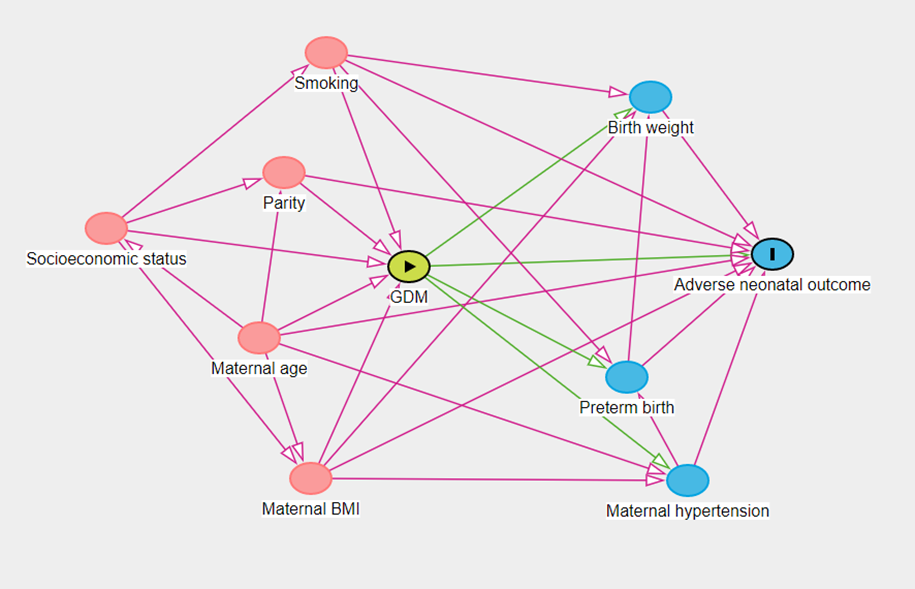


DAG, direct acyclic graph

GDM, gestational diabetes mellitus

BMI, body mass index
